# A social health services model to promote active ageing in Mexico: design and evaluation of a pilot programme

**DOI:** 10.1017/S0144686X14000361

**Published:** 2014-05-23

**Authors:** RICARDO PÉREZ-CUEVAS, SVETLANA V. DOUBOVA, LAURA ANGÉLICA BAZALDÚA-MERINO, HORTENSIA REYES-MORALES, DAVID MARTÍNEZ, ROBERTO KARAM, CARLOS GAMEZ, ONOFRE MUÑOZ-HERNÁNDEZ

**Affiliations:** * Division of Social Protection and Health, Inter-American Development Bank, Mexico City, Mexico.; † Epidemiology and Health Services Research Unit CMN Siglo XXI, Mexican Institute of Social Security, Mexico City, Mexico.; ‡ Center of Social Care for Older Adults, Mexican Institute of Social Security, Mexico City, Mexico.; § Center of Health Systems Research, Mexican Institute of Public Health, Mexico City, Mexico.; ∥ Planet Media Mexico, Mexico City, Mexico.; ¶ Social Benefits Coordination, Mexican Institute of Social Security, Mexico City, Mexico.; ** Research Division, Hospital Infantil de México Federico Gómez, Mexico City, Mexico.

**Keywords:** active ageing, pilot programme, occupational functioning, health-related quality of life

## Abstract

The objective of the study was to design and evaluate a pilot programme aimed at promoting the active ageing of older adults at the Mexican Institute of Social Security. The study was conducted in three stages: (a) design; (b) implementation; and (c) before–after evaluation through analysis of changes in functional status, occupational functioning and health-related quality of life. To overcome the limitations of the study design, we evaluated the effect of 80 per cent adherence to the programme on the outcome variables using the generalised linear regression models (GLM). Two hundred and thirty-nine older adults agreed to participate, of whom 65 per cent completed the programme. Most were women; the average age was 77 years. Adherence to the programme was higher than 75 per cent for the group who completed active ageing services and less than 60 per cent for the drop-out group. Overall, 46 per cent of older adults reached an adherence level of 80 per cent or higher. Adherence was significantly associated with improved quality of life total score (coefficient 2.7, *p*<0.0001) and occupational functioning total score (coefficient 2.2, *p*<0.0001). Participation of older adults in an active ageing programme may improve their health-related quality of life and occupational functioning. It is necessary to identify the potential barriers and to implement strategies to improve the recruitment and retention rates during the intervention.

## Introduction

The World Health Organization (2002) defines active ageing as the process of optimising opportunities for health, participation and security in order to enhance quality of life as people age. It also supports the notion that countries would be able to respond better to the health and social needs of the elderly if governments and civil society implement policies and programmes focused on active ageing.

Multiple research-based complex interventions are effective for improving physical function, maintaining independence and increasing quality of life among older adults (Beswick *et al*. 2008; Orellano, Colón and Arbesman 2012). Such interventions usually include an individual comprehensive geriatric assessment to identify health and social needs and to implement a plan tailored to meet such needs (Beswick *et al*. 2008; Burns *et al*. 2000; Caplan *et al*. 2004). The evidence is strong for multi-component occupation-based and client-centred interventions to improve and maintain older adults' performance of instrumental activities of daily living, especially when they incorporate cognitive-behavioural techniques (Arbesman and Mosley 2012; Orellano, Colón and Arbesman 2012). Psycho-social interventions focusing on enhancing the mental health of older adults, targeting meaningful social activities, and tailoring the person's abilities and preferences significantly improved positive mental health and quality of life and decreased depressive symptoms (Forsman, Nordmyr and Wahlbeck 2011). Furthermore, for the multiple-component group physical exercise significantly reduced risk and rate of falls, particularly when exercises for improving balance such as tai chi are included (Gillespie *et al*. 2010). Other activities that have shown beneficial effects for older adults are aerobic physical activities, which contribute to the improvement of cardio-respiratory fitness and consequently benefit cognitive function in older adults without known cognitive impairment (Angevaren *et al.* 2008).

Implementing active ageing activities in low- and middle-income countries (LMIC) is a challenging task. Higher-income countries begin to experience population ageing earlier than low-income nations; nonetheless, in the long run lower-income nations will experience the greatest burden associated with this demographic change (National Research Council 2001). By 2025, 70 per cent of the world's older adults will live in developing countries (World Health Organization 2002). Latin America is facing the ageing of its population at a rapid pace (Economic Commission for Latin America and the Caribbean and the United Nations Population Fund 2003). The elderly population of this region is a vulnerable segment of the population owing to a deteriorated health status as a consequence of the insufficient social and economic support they receive; this includes low pensions and unequal access to health care (Lloyd-Sherlock and Redondo 2009; Organisation for Economic Co-operation and Development 2009). Furthermore, the social and health policies and programmes for this age group are in an embryonic stage; however, these are already facing increasing financial pressures. Thus, an unmet need exists: few programmes in LMIC are aimed at improving physical functioning and maintaining independence and an active life (Mendoza-Núñez, Martínez-Maldonado and Correa-Muñoz 2009; Ministry of Health of the Nation 2007).

Mexico faces a rapid ageing of its population where there is also a high prevalence of chronic non-communicable diseases and disability. Between 2010 and 2050, the proportion of older adults will increase from 9 to 27 per cent of the total population, which currently is reported to be 112 million individuals (National Population Council 2006). The 2012 National Survey of Health and Nutrition of Mexico (National Institute of Public Health 2012) reported that 27 per cent of older adults had some degree of dependency; 30 per cent had fallen at least once in the previous 12 months, and this age group represented 30 per cent of the total number of ambulatory visits due to cardiovascular diseases and diabetes.

Research-based interventions for active ageing are incipient in Mexico. An ongoing five year-long active ageing model has been in operation in the state of Hidalgo since 2009 (Mendoza-Núñez, Martínez-Maldonado and Correa-Muñoz 2009). This study trains older adults to become gerontological promoters who act as self-help help-group co-ordinators (gerontological nuclei) and establish self-care and self-promotion actions for the wellbeing and social development of the elderly population. The model has proven to be feasible from the technical and operational perspectives; but is necessary to develop further its institutional and social feasibility (Mendoza-Nuñez and Martinez-Maldonado 2013).

The Mexican health system is segmented and the population receives health and social benefits according to their employment status. The Social Security system follows the principles of traditional social health insurance schemes in which persons employed in the formal labour market are mandated to make contributions (through a progressive payroll tax rate) in order to be entitled to receive social, economic (pensions and disability leaves) and health-care benefits for the employees and their families, including their parents. Sixty per cent of the population is affiliated with the Social Security system, whereas 40 per cent of the population depends on non-contributory social and health programmes, which are financed through general revenues.

The Mexican Institute of Social Security (IMSS) is the largest nationwide social security institution. IMSS has approximately 40 million affiliates. Health benefits are provided through a tripartite health-care system where the base of the pyramid is the primary care services. Social services are delivered through a network of social security centres mostly focused on promoting physical and cultural activities for all ages. These centres provide different activities for older adults, including physical exercise, manual arts, education for care-takers and prevention of household accidents.

The population affiliated with the Social Security system is experiencing a more rapid ageing process than the non-insured population. This demographic change is generating an increasing demand for health and social services. In 2006, the IMSS reported that older adults accounted for 12 per cent of its affiliates, more than 25 per cent of health-care services and 40 per cent of health expenditures (Mexican Institute of Social Security 2007). However, the institution recognised that most of its health-care and social services and programmes for older adults require strengthening.

Within the Mexican context, to promote person-centred active ageing is reasonable to narrow the gap between health care and social services. The organisational characteristics of IMSS allow interventions to be designed that can be integrated, fully co-ordinated and cost-effective, as the World Health Organization (2002) suggests.

The objective of the study was to design and evaluate a pilot programme for active ageing in terms of its feasibility and improvement in functional status, occupational functioning and health-related quality of life among participants. This paper presents the main results of the pilot programme and discusses its applicability to scale up the model for a full intervention that eventually can become a nationwide programme.

## Methods

The study was conducted in three stages: design, implementation and evaluation. The IMSS Institutional Review Board and Ethics Committee reviewed and approved the project. The design of the programme began in 2007 and the provision of services began in 2010.

### Stage 1: Design of the Centre of Social Health Services for Older Adults

The design, structure and processes of care of the Centre of Social Health Services for Older Adults (COASH) were based on European models of social health care (Ministry of Labour and Social Affairs, IMSERSO and Secretary of State for Social Services, Families and Disability 2004).

The structure comprised a multi-disciplinary team integrated by a geriatrician, rehabilitation specialist, dentist, psychologist, social worker, nurses, podiatrist, dietitian and an information technology educator. The structure also comprised the architectural design of the facility and the introduction of specialised equipment.

The processes of care included two core services: comprehensive geriatric assessment (CGA) and active ageing. CGA is a multi-dimensional, inter-disciplinary, diagnostic process that enables care needs to be identified and allows individualised care plans for older adults.

The CGA includes eight domains:

1. Clinical history, laboratory tests and medical diagnosis. The source of information was the electronic health record.
2. Nutritional status, which was measured through the mini-nutritional assessment tool (Guigoz 2006) and the body mass index (BMI).
3. Oral health measured through the geriatric oral health index (Atchison and Dolan 1990).
4. Functional status, measured through basic (Mahoney and Barthel 1965) and instrumental activities of daily living (Lawton and Brody 1969), and the modified Performance Oriented Assessment of Mobility Problems of Tinetti (Tinetti 1986) validated in Spain (Rodríguez-Guevara and Lugo 2012).
5. Psychological health, which included cognitive and affective status assessment using the Folstein mini-mental state examination (Beaman *et al.* 2004), Yesavage Geriatric depression scale (GDS-15) (Campo-Arias, Urruchurtu-Mendoza and Solano-Morales 2008) and a sleep hygiene questionnaire.
6. Podiatric assessment including dermatological, vascular, neurological and orthopaedic foot assessment.
7. Lifestyle assessment that comprised active smoking (number of cigarettes actually smoked per day), alcohol consumption (number of drinks per week), physical activity (type, frequency and duration) and frequency of food consumption during the last month. For this assessment, the *ad hoc* designed format based on older adult self-report was used.
8. Socio-environmental information such as social networks and support measured by the inventory of social resources of the elderly (Diaz Veiga 1987), Zarit Burden interview, Model of Human Occupation Screening Tool (MOHOST) (Parkinson, Forsyth and Kielhofner 2006) and health-related quality of life questionnaire (WHOQOL-BREF) (World Health Organization 1996).

The active ageing services included the following modules (Table 1):

• Social therapy to strengthen and promote social networks.
• Physical therapy to restore physical function and mobility, prevent potential injuries and maintain/improve fitness and health.
• Mental health with two sub-modules: psychotherapy and cognitive therapy.
• Education for self-care with two sub-modules: occupational therapy and self-care.
• Leisure time and communication with two sub-modules: education for appropriate use of leisure time and communication technologies.

There were three types of intervention: required (all participants were required to go through these interventions), selective (in accordance with the results of the CGA) and optional (free choice of the participants). The working group of the pilot programme defined the thematic content, number and duration of each module. The literature review supported the decision. Also, the potential demand due to the expected number of participants influenced the decision.

**Table 1.** Content of active ageing services

Modules	Number and duration of sessions	Type	Activities/themes and techniques
Social therapy	48 sessions, 1 hour, 30 minutes	Required; interactive theoretical/practical^1^ group sessions	Major themes: biological changes, self-esteem, empowerment, social networks, prevention of accidents including home safety assessment, elder abuse and legal rights. Techniques: cognitive-behavioural.^2^
Physical therapy	24 sessions, 1 hour	Required; interactive theoretical/practical group sessions. Two types of group sessions according to the functional capacity of older adults	1. Qigong, which includes practise of aligning breath, exercises for improving balance and meditation. 2. Multiple-component group exercise that includes aerobic physical activities, progressive resistance strength training that involves all major muscle groups and exercises for improving balance and flexibility.
Mental health with two sub-modules: A: Psychotherapy B: Cognitive therapy	24 sessions, 1 hour, 30 minutes (by sub-module)	Selective; interactive theoretical/practical group sessions A: For older adults with depression and grief B: For older adults with memory loss	A: Psycho-education aims at helping subjects to identify the contributing factors for depression and to deal effectively with the psychological, behavioural, interpersonal and situational contributors. Techniques: psycho-educational.^1^ Older adults with severe or moderate depression receive 5–8 sessions of individual brief psycho-dynamic psychotherapy before the group sessions. This psychotherapy is aimed at developing an optimal plan to resolve the patient's crisis situation through the use of an initial dynamic interpretation and its working through. B: Cognitive therapy that includes techniques for improving memory in congruence with the level of schooling and cognitive decline.
Education for self-care with two sub-modules: A: Occupational therapy B: Self-care	A: 24 sessions, 1 hour, 30 minutes B: 36 sessions, 1 hour, 30 minutes	A: Required; interactive theoretical/practical group sessions B: Selective; theoretical/practical group sessions	A: Part 1, Client-centred occupational therapy based on the model of human occupation (Kielhofner 2008). Part 2, Education for self-care: spinal health, posture and general rehabilitation of joints. B: Part 1, Nutritional counselling for chronic diseases patients (diabetes, hypertension) and nutritional disorders (undernutrition, overweight and obesity). Part 2, Self-care for diabetes and hypertension patients. Part 3, Self-care for urinary incontinence patients. Part 4, Personal foot hygiene. Part 5, Oral hygiene. Techniques: cognitive-behavioural.
Leisure time and communication with two sub-modules: A: Education for the proper use of leisure time B: Communication technologies	A and B: 48 sessions, 1 hour, 30 minutes (by sub-module)	A and B: Optional; interactive theoretical/practical group sessions	A: Education for the proper use of leisure time. B: Communication technologies to teach use of computers, internet and mobile phones. Techniques: cognitive-behavioural and recreational.

*Notes*: 1. Theoretical/practical: theory 15–25 per cent and practice 75–85 per cent of session time. 2. Cognitive-behavioural techniques to facilitate the emergence of desirable behaviours and maintain modified behaviour, such as positive communication, verbal incitement, shaping, generalisation training, reinforcement, motivation self-registration, self-evaluation, self-motivation, self-effort, resolution of problems, among others.

Individual and group active ageing interventions were tailored in accordance with the results of the CGA. A personalised list of activities and information about facilities for carrying out physical exercise was created as advice for older adults regarding how to modify health risks. An electronic geriatric social health record was designed for routine use. It included the above-mentioned domains of the CGA and individual follow-up plans of active ageing services.

### Stage 2: Implementation

The COASH is located in the north-west region of Mexico City within a social services complex affiliated with the IMSS. There are three family medicine clinics within its catchment area. The clinics provide care to approximately 350,000 people among whom 65,000 are older than 60 years of age. The family physicians working at these clinics were informed and invited to refer potential candidates who fulfilled the inclusion criteria.

Before the pilot programme was delivered, COASH personnel participated in a training course to learn the processes of care and use of the electronic geriatric social health record. The training activities lasted one month and were reinforced periodically throughout the year.

The COASH pilot programme was delivered sequentially. First, the authorities of each clinic and the family physicians were informed about the project and were invited to refer adults over 65 years of age with mild to moderate physical dependency to the Centre. Second, all older adults referred underwent the CGA. This allowed care plans to be identified and tailored for those who were fit to participate in the active ageing services. Older adults suffering from uncontrolled chronic conditions, psycho-geriatric disorders impairing functionality, falls or injuries in the last 72 hours, or those with severe physical dependency (Barthel index score for basic activities <60 points) were not considered suitable participants; instead they were counter-referred with specific recommendations to the family medicine clinic to continue their treatment.

The older adults able to participate were asked for their informed consent before being enrolled. They were then evaluated and their individual plans were developed and implemented. Each participant was expected to remain in the programme for one year, after which the second CGA was performed. Participants who presented any medical emergency requiring ambulatory or hospital care during the active ageing activities were counter-referred. At the end of the active ageing activities, all participants were counter-referred to their family medicine clinics.

The active ageing pilot programme employed currently recommended recruitment and retention strategies (Deakin University's Centre for Physical Activity and Nutrition Research 2012; Fletcher *et al.* 2012; McMahon, Talley and Wyman 2011). The recruitment strategies included providing information and reinforcement of the potential benefits of the intervention to family physicians and older adults, referrals through the family physicians and dissemination of printed materials. The retention strategies incorporated client-centred approaches and cognitive behavioural techniques to gain the commitment of older adults towards behavioural changes and adherence to the programme. Other retention strategies included opportunities for socialisation within the programme and follow-up phone calls to query the cause of non-attendance and to facilitate the participant to reschedule a new appointment.

Dropouts were those participants who failed to continue the active ageing services and did not have the post-intervention evaluation.

### Stage 3: Evaluation of the pilot programme

This was a ‘before and after’ evaluation with no control group to analyse changes in functional status, occupational functioning and health-related quality of life of participants. The source of information was the electronic geriatric social health record. This information was analysed from those participants who were recruited and completed the COASH services during the period between August 2010 and June 2012.

To describe the population, the study variables were as follows:

1. General characteristics and lifestyle: gender, age, literacy, life partner, children, friends, smoking and alcohol consumption, and regular physical activities.
2. Medical history: number and type of chronic diseases and geriatric syndromes.
3. Nutritional status: the Canadian guidelines for body weight classification in adults (Douketis *et al.* 2005) served to evaluate the nutritional status: malnutrition (BMI<18.5 kg/m^2^); underweight (18.5–21.9); normal weight (22–29.9); obesity (>30).
4. The outcome variables were measured through the changes in basic and instrumental activities, gait and balance, occupational functioning, and through the increase in regular physical exercise, and improvement in the health-related quality of life areas (Table 2).
5. Adherence to the active ageing services was defined as compliance of 80 per cent or higher of the required and selective group sessions.

**Table 2.** Study outcome variables and their assessment tools

Outcome variables	Assessment tools and evaluation criteria
Basic activities of daily living	*Barthel index* Ten basic activities of daily living are evaluated: lying down and getting of the bed, walking from the bed to a chair and *vice versa*, walking within the house, dressing/undressing, bathing, eating, using the toilet and maintaining sphincter continence. Each activity comprises five levels of dependency; the maximum score on this scale is 100 points representing independence in daily living.
Instrumental activities of daily living	*Lawton and Brody scale* Eight instrumental activities are evaluated: using the telephone, shopping, preparing food, housekeeping, doing laundry, mode of transportation, responsibility for own medication and ability to handle finances. For each activity one of three values was assigned: 0=dependent, 1=needing some help, 2=independent. Total score is 16 with higher scores reflecting greater independence in instrumental activities.
Gait and balance	*Modified Performance Oriented Assessment of Mobility Problems of Tinetti* The overall mobility scores range from 0 to 28, with a higher score (25–28 points) indicating a low risk of falls.
Occupational functioning	*Model of Human Occupation Screening Tool (MOHOST)* ‘This tool determines the extent to which client factors (values, personal causation, interests, roles, habits and skills) and environmental factors (physical and social) facilitate or restrict an individual's participation in daily life.’ MOHOST has six domains: (a) volition (motivation for occupation); (b) habituation (pattern of occupation); (c) communication and interaction skills; (d) process skills; (e) motor skills; and (f) environment. Each domain has four items and the score per domain ranges from 4 to 16. The total score ranges from 24 to 96; the higher the score, the better the occupational functioning.
Regular physical activities	*Self-report* Regular physical activity was defined if older adult participates in at least 150 minutes of moderate-intensity aerobic activity during the previous three months. This definition was based on the ‘Global recommendations on physical activity for health’ by the World Health Organization (2010).
Health-related quality of life	*WHOQOL-BREF* Comprises physical and psychological health, social relationships and environment, overall perception of quality of life and general health. The crude results should be standardised to a weighted scale from 0 to 100. The higher the score, the better the perception of health-related quality of life.

### Sample size

The sample size was calculated to detect the minimum difference between the final and baseline measurement of 3 points on the social domain of the quality of life scale: 73.9 (standard deviation (SD): 16.9) at baseline (Gonzales-Celis and Padilla 2006) and 76.9 (SD 16.9) at the final evaluation. With an alpha of 0.05 (one-sided) and beta of 0.85, the sample size should be 229. Considering 10 per cent of the possible loss during follow-up, the sample size should be 252. The sample size for a one-tailed paired-sample *t*-test was calculated using the Stata statistical package. We chose this outcome variable because it allows comprehensive evaluation of the pilot programme.

### Statistical analysis

The before and after evaluations were conducted by comparing the mean differences (including the estimate of the 95 per cent confidence intervals (CI)) of the outcome variables using the paired-sample *t*-test. The analysis was performed according to the intention-to-treat approach. Missing measurements due to a participant's withdrawal from the study were replaced with the most recent observations (*i.e.* last observation carried forward). The analysis included a comparison of general characteristics between those older adults who completed the intervention and those who dropped out. These comparisons were made using a chi-squared test for categorical variables and Student's *t*-test for independent samples for continuous variables.

To evaluate the effect of adherence (80 per cent or more) to the active ageing services on the outcome variables, we built GLM. The GLM method considers the correlation among repeated observations of the same subject and allows a more precise estimate to be obtained (Liang and Zeger 1986). Each outcome variable was analysed separately; thus, in every GLM the dependent variable was one of the outcome variables (endline evaluation), and the independent variable was adherence. Each GLM was adjusted for clinically relevant covariates. The association between adherence and functional status was adjusted for gender, age, chronic disease, and the baseline results of regular physical activities and functional status. The association between adherence and occupational functioning was adjusted for gender, age and baseline results of the occupational functioning variables. The association between adherence and quality of life was adjusted for gender, age, educational level (primary school or higher), chronic disease, and baseline evaluation of depression, regular physical activities and quality of life. The association between adherence and regular physical activity was adjusted for gender, age, chronic disease and baseline evaluation of depression and regular physical activities; *p*<0.05 was considered as statistically significant. The analysis was performed using Stata 12.0 statistical software (Stata/SE 12.0, Stata Corporation, College Station, Texas, USA).

## Results

**Figure 1.**
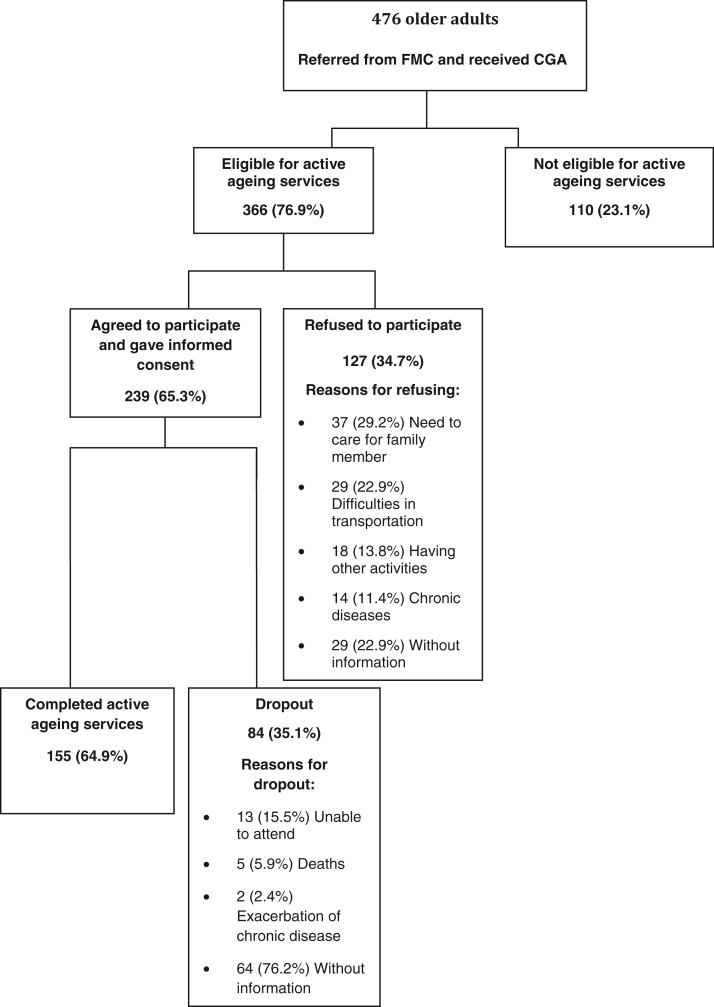
Flow of participants.

*Notes*: FMC: family medicine clinic. CGA: comprehensive geriatric assessment.

Figure 1 describes the flow of participants who attended COASH between August 2010 and June 2011; 476 older adults underwent CGA, and 77 per cent of these were candidates for receiving active ageing services. Among the eligible candidates, 66 per cent agreed to participate. Among those who agreed, 65 per cent completed the pilot programme.

Table 3 shows the general characteristics, lifestyle and nutritional status of the older adults who completed the active ageing services and the dropouts. Most participants were women with a median age of 77 years. In the group of dropouts there were more males, and they were older than the other groups (*p*<0.05). In both groups almost 40 per cent of the participants were illiterate or had not completed primary school. Half of them were living with their life partner, although in the dropout group this percentage was higher (68%, *p*<0.05). Most had children and friends. Regarding lifestyle, those who completed the intervention smoked less often (*p*<0.05) and practised regular physical activities more often than the other group (*p*>0.05). Regarding nutritional status, on average 5 per cent were underweight, 61 per cent were normal weight and 33 per cent were obese; no statistical differences were observed between groups.

**Table 3.** General characteristics, lifestyle and nutritional status

Variables	Completed active ageing services	Dropout	Total
	*Percentages*		
Female*	74.8	54.8	67.8
Age (years):*			
Median	76	78	77
Minimum–maximum	66–87	67–97	67–97
Primary school or higher	58.1	60.7	59.0
Living with life partner*	51.0	68.7	67.1
Having children	99.4	97.6	98.7
Having friends	78.7	68.7	75.2
Smoking*	3.2	7.1	4.6
Alcohol consumption	7.1	9.5	7.9
Regular physical activities	33.5	28.6	31.8
Nutritional status:			
Malnutrition	0	2.4	0.8
Underweight	4.5	3.6	4.2
Normal weight	58.7	60.7	59.4
Obese	36.8	33.3	35.6
N	155	84	239

*Significance level*: * *p*<0.05 (between groups).

Table 4 shows the medical conditions of the two groups. Almost all suffered from two or more chronic conditions such as musculoskeletal disorders followed by cardiovascular diseases. One-third of the participants suffered from gastrointestinal conditions, 25 per cent had diabetes and 19 per cent had neoplasms; 88 per cent had at least one geriatric syndrome such as sensory deprivation (59%), urinary or faecal incontinence (46%), depression (34%) or insomnia (29%). The dropout group suffered falls more often (29.8%) than those who completed the active ageing services (16.8%, *p*<0.05).

**Table 4.** Medical history

Variables	Completed active ageing services	Dropout	Total
	*Percentages*		
Chronic diseases:	97.4	97.6	97.5
Median number	2	2	2
Minimum–maximum number	0–5	0–5	0–5
Cardiovascular diseases	71.0	71.4	71.1
Musculoskeletal disorders*	75.5	64.3	71.5
Gastrointestinal diseases	29.0	31.0	29.7
Diabetes	23.9	28.6	25.5
Other endocrine diseases	3.2	2.4	2.9
Neoplasms	16.8	25.0	19.7
Respiratory tract diseases	7.7	9.5	8.4
Geriatric syndromes:	90.3	83.3	87.9
Median number	2	2	2
Minimum–maximum number	0–6	0–5	0–6
Sensory deprivation	62.6	52.4	59.0
Urinary and/or faecal incontinence*	54.2	32.1	46.4
Depression	36.1	30.5	34.2
Insomnia*	34.8	20.2	29.7
Falls*	16.8	29.8	21.3
Constipation	18.7	10.7	15.9
Orthostatic hypotension*	18.1	2.4	12.6
Pain syndrome	9.7	11.9	10.5
Cognitive impairment	5.2	3.6	4.6
Syncope	0.6	1.2	0.8
N	155	84	239

*Significance level*: * *p*<0.05 (between groups).

Adherence to the required and selective modules of the programme was higher than 75 per cent for the group who completed active ageing services (mean adherence ranged from 72% for cognitive therapy to 89% for occupational therapy modules) and less than 60 per cent for the dropout group (mean adherence ranged from 58% for social therapy to 74% for occupational therapy modules). Overall, 46.1 per cent of older adults reached an adherence level of 80 per cent or more to the active ageing services (data not presented in the tables).

Table 5 depicts the changes in the outcome variables and the association between adherence to the programme and the end-line scores of these variables. The programme contributed to improving occupational functioning, in particular the domains of volition, process skills and habituation (*p*<0.05). It also improved the perception of quality of life. The greatest change was in the environment domain (3.4, 95% CI: 2.4–4.4, *p*<0.0001) followed by the psychological and social relationships domains. The percentage of those who practised regular physical activity increased significantly (from 33.5 to 59.4%, *p*<0.001).

**Table 5.** Changes in functional status, occupational functioning and health-related quality of life at the end of the active ageing service interventions of the Centre of Social Health Services for Older Adults (COASH) and the association between adherence to the programme and end-line scores in these variables

	Baseline (mean and SD)	End-line (mean and SD)	Changes between end-line and baseline (mean and 95% CI)	Association between ⩾80% adherence to the programme and outcome variables^1^	
				*β* adjusted (95% CI)	*p*
Functional status:					
Basic activities	96.3 (6.3)	96.0 (7.2)	−0.3 (−0.8, 0.2)	−0.1 (−1.1, 0.8)	0.772
Instrumental activities	13.08 (3.3)	13.12 (3.2)	0.04 (−0.16, 0.24)	0.03 (−0.36, 0.43)	0.868
Overall mobility score	21.4 (3.8)	21.8 (3.1)	0.4 (0.2, 10.7)*	0.3 (−0.16, 0.70)	0.216
Occupational functioning:					
Volition	12.4 (4.0)	13.6 (2.7)	1.2 (0.8, 1.96)*	**0.7 (0.2, 1.3)**	**0.007**
Habituation	12.9 (3.4)	13.9 (2.2)	1.1 (0.7, 1.4)*	**0.7 (0.2, 1.2)**	**0.006**
Communication and interaction skills	13.2 (3.4)	14.0 (2.6)	0.8 (0.4, 1.2)*	0.3 (−0.2, 0.9)	0.260
Process skills	12.7 (3.6)	14.2 (2.6)	1.4 (1.0, 1.8)*	0.3 (−0.3, 0.9)	0.388
Motor skills	12.1 (3.9)	12.9 (3.5)	0.8 (0.5, 1.1)*	0.03 (−0.5, 0.6)	0.923
Environment	12.8 (3.6)	13.8 (2.8)	0.9 (0.6, 1.3)*	−0.05 (−0.6, 0.4)	0.823
Total score	76.1 (20.2)	82.3 (14.6)	6.2 (4.7, 7.6)*	**2.2 (0.1, 4.3)**	**0.039**
Quality of life:^2^					
Physical health	75.0 (8.8)	75.6 (9.4)	0.5 (−0.4, 1.5)	**0.9 (−0.9, 2.8)**	**0.304**
Psychological	70.8 (8.5)	73.4 (8.3)	2.6 (1.5, 3.6)*	**4.3 (2.4, 6.2)**	**0.0001**
Social relationships	76.3 (8.6)	78.1 (8.5)	1.9 (0.8, 2.9)*	**3.9 (5.1, 2.7)**	**0.0001**
Environment	68.2 (7.2)	71.7 (7.7)	3.4 (2.4, 4.4)*	**3.0 (1.3, 4.7)**	**0.0001**
Total score	66.3 (5.2)	68.3 (5.8)	2.0 (1.3, 2.7)*	**2.7 (1.5, 3.9)**	**0.0001**
Regular physical activities (%)	33.5	59.4	26.8 (26.72, 26.87)*	**1.4 (0.8, 2.1)**	**0.0001**

*Notes*: N=239. SD: standard deviation. CI: confidence interval. 1. The effect of ⩾80% adherence to COASH in generalised linear regression models with end-line evaluations adjusted for clinically important covariates. The bold values highlight the statistically significant adjusted coefficients. 2.

*Significance level*: * *p*<0.001 (between the end-line and baseline measurements).

Adherence was significantly associated with improved quality of life total score (coefficient 2.7, *p*<0.0001) after adjusting for other covariates; in particular, it increased the following domains of quality of life: psychological (coefficient 4.3, *p*<0.0001), social relationships (coefficient 3.9, *p*<0.0001) and environment (coefficient 2.7, *p*<0.0001). Also, we observed a positive association between adherence and the occupational functioning total score (coefficient 2.2, *p*<0.0001), which included the volition and habituation domains (coefficient 0.7, *p*<0.007). Additionally, the association between adherence and regular physical activity of older adults (coefficient 1.4, *p*<0.0001) was positive. The associations between adherence and other outcome variables were not statistically significant.

## Discussion

The main findings of this study point out that the implementation of the COASH active ageing pilot programme faced challenges for recruitment and retention, but also achieved beneficial effects regarding occupational functioning, health-related quality of life and regular physical activity of participants.

The pilot programme had a moderate percentage of acceptance (66%) and retention rate (65%) which were below those reported in similar studies. Previous interventions on older adults reported difficulties in recruitment and retention (Jancey *et al.* 2006; Nyman and Victor 2012). A recent Cochrane review of 99 falls prevention interventions reported that the median recruitment rate was 70.7 per cent (64.2–81.7%, N=78 studies) and the median attrition rate at 12 months was 10.9 per cent (9.1–16.0%, N=44 studies) (Nyman and Victor 2012). Adherence to multifactorial interventions was generally higher than or equal to 75 per cent but ranged from 28 to 95 per cent for individual components. This Cochrane review proposed that the design of future interventions to reduce falls in community-dwelling older adults should anticipate, on average, a recruitment rate of 70 per cent, dropout rate of 10 per cent at 12 months and an 80 per cent adherence rate during the intervention.

The socio-demographic conditions of participants had an influence on recruitment and retention. In this pilot programme it was more difficult to recruit and retain male than female older adults, as well as those who were oldest, came from low socio-economic groups had a low level of literacy and unhealthy behaviours. Other studies reported similar findings (Arean and Gallagher-Thompson 1996; Eakin *et al.* 2007; O'Neill *et al.* 1995). To increase the recruitment and retention rates, additional strategies should be implemented to overcome the barriers. Such strategies might include revision of the age criterion for programme inclusion, provision of information congruent with the literacy level of potential participants, easing the barriers for transportation and implementing home visits to enhance adherence.

Approximately six million older adults are affiliated with the IMSS and require innovative social and health-care services. It is reasonable to take a step-by-step approach that includes the rigorous design and evaluation of new services before launching full-scale programmes that represent a significant investment for complex institutions in charge of providing social and health-care services for large groups of people.

The main components of COASH are CGA and active ageing services, which complement each other. The results of the CGA showed that most participants suffered from at least one chronic condition and/or geriatric syndrome that could benefit from receiving active ageing services. CGA has been found to be beneficial in hospitalised patients because its results guided the implementation of individualised interventions which, in turn, contributed to improving physical and cognitive functions and reducing mortality (Ellis *et al.* 2011; Stuck *et al.* 1993; Van Craen *et al.* 2010). In the present study, CGA helped to guide the individual active ageing interventions.

Participating in active ageing services has positive effects on occupational functioning. In this study, an increase was observed in the domains of motivation for occupation and habituation, which indicates obtaining positive changes concerning perceptions of self-competencies and systems of values and interests. The importance of the finding lies in the evidence that improved social functioning yields the same benefits for survival as physical activity (Glass *et al.* 1999) and is associated with better mental capacity (Wang, Karp and Winblad 2002) and quality of life (Dahan-Oliel, Gélinas and Mazer 2008).

Our active ageing services programme included physical activity as a required module because multiple studies found that physical activity is effective for preventing and treating various medical conditions in older adults, including physical and cognitive decline (Angevaren *et al.* 2008; Ashworth *et al.* 2005; Liu and Latham 2009). More participants reported practising regular physical activities at the end of the study.

The COASH pilot programme improved the quality of life of older adults. Quality of life is a particularly valuable proxy of the success of active ageing services. The World Health Organization promotes a holistic perspective of wellbeing and supports that the main objective of health care is to improve quality of life. In Mexico, use of the WHOQOL-BREF is incipient despite being a widely used instrument. A study conducted in Mexico City on 194 older adults with an average age of 71 years reported that the highest score was for the ‘social relationships’ domain (73.9 points) and the lowest was for the domain ‘physical health’ (70.3 points) (Gonzales-Celis and Padilla 2006). The main domains of improvement after participating in the COASH active ageing services were environment, psychological and social relationships; however, the final score in the ‘environment’ domain was lower than in high-income countries such as Australia (75.1 points), Denmark (74.0) and Canada (80.0) (Noerholm *et al.* 2004; Paskulin and Molzahn 2007).

Older adults are a substantial part of the society, and the notion that ageing is a burden is gradually disappearing; in accordance, the concept of active ageing focuses on maintaining participation of older adults in society regardless of their health status (Jacobs 2005). Boudini (2013) proposes centring active ageing in three key principles: fostering adaptability, supporting the maintenance of emotionally close relationships and removing structural barriers related to age or dependency. The COASH active ageing pilot programme considered these principles, through offering interactive theoretical/practical group sessions in each of the five modules (social therapy, physical therapy, mental health, education for self-care, and leisure time and communication technologies). These modules aimed to help older adults to accept age-related changes, providing them with tools to compensate for certain functional limitations and find new ways to remain engaged.

There is a wide variation in the advancement of the supply of social and health-care services for older adults in Latin America (Huenchuan 2010). In most cases, different institutions provide these services in a separate way and with little interaction between each other (Huenchuan 2010). From this perspective, social and health-care services must be interconnected to provide active ageing services. The present study illustrates the operationalisation of a model of active ageing services in a public institution of a middle-income country that interconnected both social and health-care services. The 2002 Plan of Madrid for International Action on Ageing (United Nations 2002) had an impact on the Latin American region. This programme fuelled the development of policies and programmes on active ageing in Brazil, Chile, Mexico and Argentina (Huenchuan and Rodríguez-Piñero 2011; Mendoza-Núñez, Martínez-Maldonado and Correa-Muñoz 2009; Ministry of Health of the Nation 2007). The existing literature addresses the description of such policies and programmes, but little is known about the results of the evaluations or lessons learned during its implementation. Furthermore, in the region, the design and introduction of new services and programmes for older adults within public institutions is challenging. The main barriers are resource constraints and competition, organisational hurdles, and uncertainty regarding sustainability and lack of evidence-based intervention models (Huenchuan and Rodríguez-Piñero 2011).

In terms of limitations, in this pilot programme the effect of active ageing services was evaluated by using a ‘before–after design’ with no control group. To overcome such limitations, we evaluated the effect of 80 per cent adherence to the intervention on the outcome variables using the GLM. We also ensured that the ‘before and after’ measurements were performed using the same methodology. To deal with the dropout threat to internal validity, statistical analysis was performed according to the intention-to-treat approach.

An impact assessment of COASH using a randomised experimental design is desirable. A substantial effort should be directed towards evaluating cost-effectiveness, sustainability and reproducibility of this model of services. This would help the decision-making process to institutionalise active ageing services. Another limitation is that we did not carry out a follow-up on the subjects who received CGA; however, in view of their conditions they were counter-referred to the family medicine clinics. It would be relevant to learn whether health-care personnel at the clinics implemented the recommendations resulting from the assessment.

The key messages of this study are the following:

1. In most developing countries, older adults are considered vulnerable, given their poor health status and social conditions, and programmes for them are incipient and face multiple challenges; thus it is important to propose and test models of health and social care aimed at active ageing.
2. There are challenges during the design and implementation of active ageing services that should be identified before launching a full-scale programme in an institutional setting. It is advisable to define and establish strategies to mitigate potential barriers and increase recruitment and retention rates for this type of programme.
3. Participating in an active ageing programme may improve occupational functioning, health-related quality of life and regular physical activity.
4. From the modern perspective of social and health policies for older adults, the evolving concept of active ageing should be integrated into programmes that aim at maintaining participation of older adults in society instead of considering them as a passive group.
